# Design and Optimization of the Local Laser Treatment to Improve the Formability of Age Hardenable Aluminium Alloys

**DOI:** 10.3390/ma13071576

**Published:** 2020-03-29

**Authors:** Antonio Piccininni, Gianfranco Palumbo

**Affiliations:** Department of Mechanical Engineering, Mathematics & Management Engineering, Politecnico di Bari, Via Orabona, 4, 70125 Bari, Italy; gianfranco.palumbo@poliba.it

**Keywords:** age hardenable aluminium alloy, finite element method, material characterization, multi objective optimization, deep drawing process

## Abstract

The research of innovative methodologies to improve the Aluminium alloys formability at room temperature still remains an open question: the local modification of the material properties via short-term heat treatments followed by the stamping at room temperature is reported to be an effective alternative to the forming in warm conditions. In the present work, such a methodology has been applied to the deep drawing of an age-hardenable Aluminium alloy (AA6082-T6) using an experimental/numerical approach. A preliminary extensive material characterization was aimed at investigating the material behaviour: (i) in the as-received condition (peak hardening), (ii) in the supersaturated condition (obtained by physical simulation) and (iii) after being locally solutioned via laser heating. A Finite Element based approach (Abaqus CAE, v. 6.17) was then used to design the laser treatment of the blanks to be subsequently deep drawn at room temperature: a 2D axisymmetric model of the deep drawing process was coupled with the optimization platform modeFRONTIER in order to define the radial extent of the laser heat treated area able to maximize the Limit Drawing Ratio. The experimental tests were finally conducted for validation purposes and revealed the effectiveness of the adopted approach which allowed to improve the drawability of more than 20% with respect to the as received condition (T6).

## 1. Introduction

The demanding reduction of harmful emissions from mass transportation means has been pushing the big brand manufacturers to accept the challenge of implementing new criteria for the vehicle design: beside the downsizing of the installed power (in combination with hybrid propulsion configurations), the reduction of the sprung masses of the structural components by massively using light materials, like the Aluminium (Al) alloys [1], is widely recognized as the most promising solution to maintain high performances of the vehicles [2,3] and ensure, at the same time, a high level of the passengers’ safeness [4,5], which is a key aspect especially for railway applications [6]. On the other hand, the main limitation hindering the broader implementation of Al alloys is the poor formability at room temperature, which implies the reduction of the geometrical complexity achievable in one step processing, the subsequent re-design of the stamping operations according to a multi-step scheme or the manufacturing of separate sub-parts to be subsequently welded.

The scientific research, over the last twenty years, has demonstrated that increasing the working temperature has a beneficial effect on the Al alloys formability: uniaxial tensile tests carried out on both 5xxx and 6xxx series specimens [7] reported an increase of the total elongation according to temperature (usually below the recrystallization temperature). Such a scenario opened the way to the development and optimization of new technological solutions for the sheet metal forming processes [8]. In the case of the deep drawing [9], the heating of the peripheral region of a circular blank combined with the action of a cooled punch can create a gradient of material properties able to counterbalance the stress state which arises in the material during the forming operation. It was then demonstrated that optimizing both the heating temperature and the punch speed (which is directly correlated to the strain rate the material is subjected to), the Limit Drawing Ratio (LDR) can be improved by more than 40% (up to 2.85). The increase of the working temperature is reported to be beneficial also when applied to more innovative forming processes, as in the case of the hydroforming [10,11]. Complex sound components in AA6061—as in the case of a fuel cell’s bipolar plate—could be manufactured setting the optimal temperature and oil pressure rate. Similar results were obtained for the production of a fast hardenable AC170PX benchmark component, demonstrating how the same parameters had a remarkable influence on the blank capability to fill the die cavity [12].

It is worthy of notice that the evident advantages of the warm conditions are partially counterbalanced by the need of a more complex equipment: for this reason, alternative approaches have been proposed, for example by creating in the material the suitable distribution of properties before the stamping process and, in particular, by preliminary local heat treatments in any specific areas (according to the component to be manufactured). In such a way the subsequent forming operation can be carried out at room temperature (thus using standard tools) [13]. The local modification of the material properties has been extensively studied in the case of age hardenable Al alloys (as those belonging to the 6xxx series). In fact, according to the well-known precipitation sequence of Al-Mg-Si alloys [14,15,16,17,18,19], the heat treatment can be designed to dissolve precipitates at the grain boundaries (from a T6/T4 state) to improve the material formability or, alternatively, start from a more formable condition and use the local treatment to promote the precipitation of hardening phases. In particular, due to the complexity of the process and the wide number of parameters involved in both steps, the process design is not trivial and a Finite Element (FE) based approach represents an efficient tool able to provide the optimal value of the process parameters to effectively treat selected areas while creating a very small transition region (due to the high material conductivity) [20].

In the present work, the local heat treatment approach has been applied to the deep drawing process. It is one of the most conventional sheet-metal forming processes and it is also frequently used to evaluate the material formability. The variation of the mechanical properties was achieved applying a laser treatment, able to quickly heat and cool the material in a narrow area whose shape can be easily reconfigured according to the geometry of the component or the heating strategy. By changing the heating treatment pattern, the shape accuracy can be affected and both the root mean square (RMS) deviation and the springback reduced up to about 27% [21]. Alternative solutions offered by conduction or inductive heating devices are more rigid and invasive, being created according to a specific geometry and acting in areas (for example 80 mm square conductive plates in the work of Kahrimanidis et al. [22]) larger than a laser spot (usually in the range 10–20 mm [23]).

The alloy under investigation—the age hardenable AA6082-T6—was characterized: (i) in the as received condition (peak hardening), (ii) in the supersaturated condition (calibrated by physical simulation) and (iii) after being locally solutioned via laser heating. A Finite Element based approach (Abaqus CAE, v. 6.17) was then used to design the laser treatment of the blanks and the subsequent deep drawing at room temperature. To improve the accurateness of the FE model, the unknown quantities (the coefficient governing the heat exchange between the blank and the surrounding environment during the laser heating or the friction coefficient in the case of the deep drawing simulations) were inversely determined, being the inverse analysis approach is a valuable solution to evaluate unknown and, in some case, difficult to measure quantities [24,25]. In addition, the 2D axisymmetric model of the deep drawing process was coupled with the optimization platform modeFRONTIER in order to define the radial extent of the laser heat treated area able to maximize the Limit Drawing Ratio. The experimental tests were finally conducted for validation purposes.

## 2. Material and Methodologies

### 2.1. The Investigated Alloys

The investigations in the present paper were conducted on the aluminium alloy AA6082-T6 (the chemical composition is reported in Table 1). It is an age hardenable alloy, received in the condition T6 (solution heat treated and then artificially aged) which is characterized by the maximum strength and a very limited elongation.

### 2.2. Material Characterization

In order to determine the material behaviour in the two conditions (peak hardening and supersaturated) it was necessary to: (i) tune the heat treatment for solutioning the alloy; (ii) conduct tensile tests.

#### 2.2.1. Tuning of the Solution Heat Treatment

**Figure 1:**
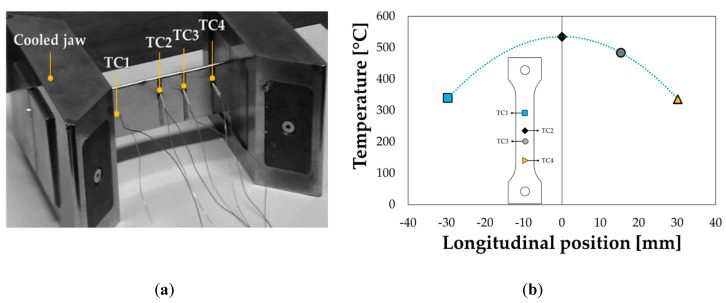
Solution Heat Treatment (SHT) tests: (**a**) thermocouple positioning; (**b**) resulting parabolic temperature distribution.

The heating cycle to get the supersaturated condition (Solution Heat Treatment, SHT) was precisely tuned by physical simulation. Data available in literature report several combinations of the parameters (heating temperature and holding time) for the SHT [26,27,28,29,30,31,32,33]. Regardless of the specific values, the heating cycle is mainly based on a preliminary heating step (heating rate in the range of 5 °C/s), a holding step (soaking time to completely bring the material in the monophasic alpha region [34]) and a cooling step (it has to be drastic to freeze the Super Saturated Solid Solution, SSSS). The SSSS condition is energetically unstable (Si has a very limited solubility in the Al matrix at room temperature [34]) and tends toward a more favourable one promoting the precipitation of secondary phases, i.e., triggering the natural ageing phenomenon [35]. In order limit the number of experimental tests, the SHT was tuned using the Gleeble 3180 physical simulator, able to create in the laboratory any thermomechanical condition on the material by means of a closed loop Joule heating system, an air/water cooling system and a hydraulic circuit for tensile load application. Tests performed in the present investigation were conducted on striped AA6082-T6 specimens (length: 120 mm, width: 30, thickness: 1.5 mm). The current able to heat up the material was modulated according to the temperature acquired by the thermocouple welded in the middle of the specimen (TC2 in Figure 1a).

Additional thermocouples were welded 15 mm (TC1) and 30 mm (TC3 and TC4) away from TC2 (on opposite sides) in order to acquire the temperature distribution. Being the ends of the specimen were clamped between cooled jaws, a parabolic distribution of temperature was obtained (Figure 1b) and maintained for the whole heating test.

#### 2.2.2. Uniaxial Tensile Tests

**Figure 2:**
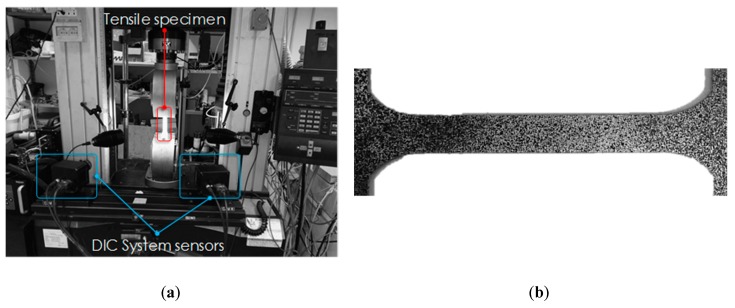
Tensile test: (**a**) experimental equipment, (**b**) detail of the speckled specimen gauge length.

Uniaxial tensile tests were carried out on the Instron 4485 universal testing machine, assisted by the Digital Image Correlation (DIC) system ARAMIS (provided by GOM) to continuously acquire the strain distribution during the test: the experimental setup is reported in Figure 2a.

Before the tensile test, the specimens were sprayed with a first white matte layer to avoid reflections and then superimposing a random distribution of small black areas, so that the resulting pattern (speckle) could thus be recognized by the two DIC cameras as a virtual grid (Figure 2b).

#### 2.2.3. Post-Treatment Material Behaviour at room Temperature

**Figure 3:**
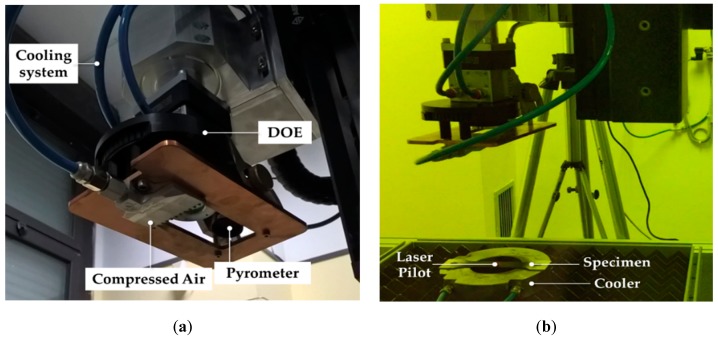
Laser Heat Treatment (LHT) tests on AA6082-T6 tensile specimens: (**a**) detailed view of the laser heating head, (**b**) experimental setup.

The investigation of the material behaviour was completed by performing a Laser Heat Treatment (LHT) in the central area of the dog-bone specimen. Once cooled down at room temperature, it was subjected to the tensile test. The laser treatment was performed using the 2.5 kW CO_2_ Rofin DC025 source and the equipment shown in Figure 3a, consisting of the Diffractive Optical Element (to change the power distribution from Gaussian into top hat), the lens (20 mm square spot), the pyrometer and the cooling system.

As shown in Figure 3b, the sample to be treated was placed on a ring-shaped metallic support to prevent excessive heat conduction to its ends. 

Due to the elevated reflectivity of the Al alloy, the portion of the specimen to be treated was sprayed with a black paint to improve the absorption coefficient. In addition, a wire thermocouple was welded in the middle of the specimen on the side not irradiated by the laser beam.

According to the methodology proposed in literature [23], the material properties were changed using the LHT. In particular, in order to reach the heat treatment conditions determined through the physical simulation, the laser power was set to 750 W and the holding time to 5 s. The adopted laser parameters appear to be coherent with the ones in literature [13], even though in the paper of Geiger et al. a Nd:YAG laser source was used; in fact, in the investigation conducted by the authors the surface of the blanks was properly coated with a graphite-based paint in order to improve the absorption coefficient.

#### 2.2.4. Formability Tests

**Figure 4:**
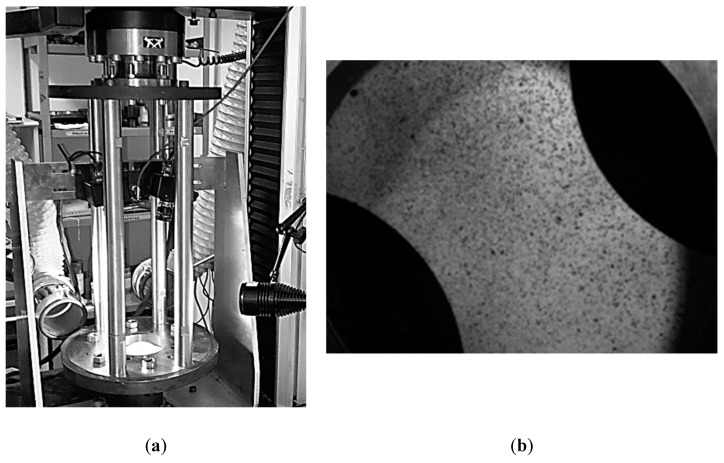
Formability tests: (**a**) the adopted equipment; (**b**) stochastic pattern for the Digital Image Correlation (DIC) analyses.

The strain behaviour of the Al alloy in the two investigated conditions was assessed by means of Nakazima formability tests adopting the equipment shown in Figure 4a, specifically designed to be: (i) assembled on the Instron 4485 universal testing machine and (ii) integrated with the ARAMIS DIC system.

As visible in Figure 4b, the specimens were painted to obtain a stochastic pattern of randomly distributed black dots on a white background in order to be processed by the DIC system.

### 2.3. Process Design using a Numerical Approach

Both the local heating and the forming operation were designed using an FE based approach.

#### 2.3.1. FE model of the Laser Heat Treatment

The experimental setup shown in Figure 3b was simulated to tune the FE model via pure thermal transient analyses: the dog-bone specimen was modelled as a 3D deformable body and meshed with 1 mm linear hexahedral elements (type DC3D8); the thermal properties of the alloy were taken from the literature [36]. In order to make the thermal simulation more accurate, two different Heat Transfer Coefficients (HTCs) were specified, the first one governing the heat exchange between the specimen and the surrounding environment and the second one between the specimen and the cooler, thus assuming the whole exchange as convective. Data from the LHT performed on dog-bone specimens were used useful to tune the FE model for thermal simulations. In particular, the main parameters affecting the thermal simulation—i.e., the two abovementioned HTCs and the absorbed portion of the irradiated energy (absorption coefficient)—were determined using an inverse analysis approach. The parameters to be tuned were iteratively changed and the discrepancy between the experimental temperature evolution and the one calculated by the numerical model in a point of interest was evaluated for each iteration (i.e., each set of values adopted for the heat transfer coefficient and the absorption coefficient).

Preliminary simulations were conducted in order to evaluate the effect of the radiation losses on the temperature distribution: in particular, simulations conducted setting the laser power at 500 W and the feed rate at 1.6 mm/s without and with considering the surface radiation (emissivity equal to 0.9) revealed an average percentage difference between the temperature distributions at different instants lower than 6%. In addition, it should be considered that, due to the tuning using experimental data (coming from both thermocouples and thermal camera acquisitions), the adopted value of the Heat Transfer Coefficient can be considered able to perfectly reproduce the whole heat transfer.

The model parameters tuned by inverse analysis could be used to design the circular laser heating of the specimens along its periphery. In particular, FE simulations, arranged according to a Full Factorial plan, were aimed at choosing the optimal laser Feed Rate (FR) and Laser Power (LP) able to effectively change the blank peripheral region into the SSSS condition.

#### 2.3.2. FE Model of the Deep Drawing Operation

**Figure 5:**
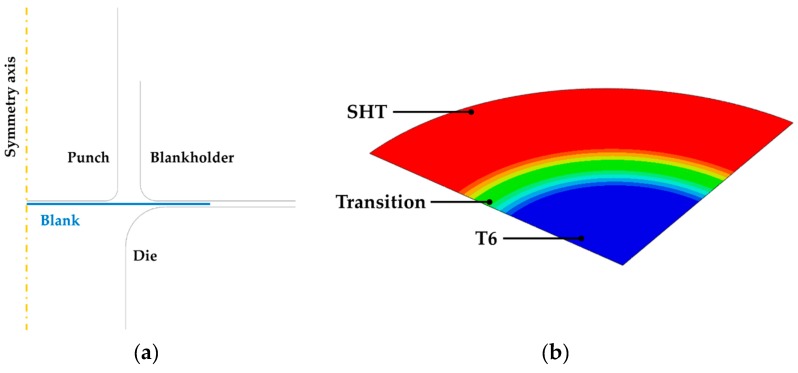
Finite Element (FE) model of the deep drawing process: (**a**) 2D axisymmetric approach, (**b**) distribution of material properties over the LHT blank.

The tests using the laboratory scale equipment (detailed in Section 2.4.2) were simulated by means of the 2D axisymmetric model shown in Figure 5a. The tools (blankholder, die and punch) were modelled as analytical rigid bodies (no meshing was needed) while the blank as a deformable shell body meshed with 0.5 mm SAX1 element (2 nodes linear axisymmetric shell element). The blank properties were modelled using a field variable: the Al alloy in the SSSS condition was assigned to the peripheral region, while the Al alloy in the T6 condition was assigned to the remaining portion of the blank. A linear transition region (extent: 10 mm, according to the results from the material characterization) was modelled between the two regions.

The experimental Forming Limit Curves (FLCs) concerning both the investigated conditions were implemented to activate a damage criterion; for the sake of clarity, the modelling of the material distribution resulting from the LHT process adopting a field variable is depicted with a 3D representation (one quarter of the whole blank) in Figure 5b. The plastic behaviour was modelled according to the isotropic Von Mises yield formulation. The contact between the blank and the punch was modelled according to a penalty formulation, setting the coefficient of friction between the blank and the punch equal to 0.2 [37,38,39], whereas the coefficient of friction of the other contact pairs (blank/die and blank/blankholder) was defined using the inverse analysis based on the minimization of the error between experimental data (punch load-stroke curve) and the correspondent numerical simulation. 

#### 2.3.3. Process Optimization using the Genetic Algorithm

The 2D axisymmetric model, being the computational cost of a single run in the order of few minutes, was considered particularly suitable to be coupled with the optimization platform (modeFRONTIER). In particular, an automatic procedure was set up in order to optimize the extent of the blank portion to be fully solutioned thus obtaining the highest value of the LDR. The initial blank radius (R) and the extent of the portion in the T6 condition (L_T6_ and expressed as a percentage of the initial blank radius), being the input variables of the problem, have been reported in Table 2 together with their variation ranges.

The maximum value of the damage variable FLDCRT (which measures how close the strain condition is to the FLC data) was used to evaluate not only the occurrence of the fracture but also to judge how much critical the strain condition is: the objective function was thus based on the minimization of the FLDCRT variable. An initial population (20 designs) was created adopting the Sobol algorithm, a deterministic sequence characterized by a sensible reduction of the clustering effect and able to fill the design space in a uniform way [40]. The multi-objective genetic algorithm MOGA-II was used for driving the optimization loop, being such an algorithm characterized by the smart multi-search elitism able to preserve few excellent solutions without causing premature convergence to local optima [41,42]. The number generations performed was set to 20, thus a total number of 400 designs were analysed (initial population multiplied by the number generations).

### 2.4. Experimental Activity

#### 2.4.1. Laser Heat Treatment of the Circular Blanks

**Figure 6:**
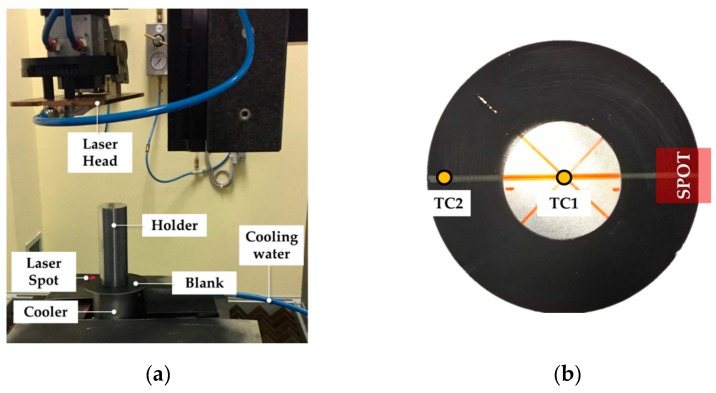
Local laser treatment: (**a**) 102 mm diameter blank preparation, (**b**) experimental setup.

The adopted experimental set up is shown in Figure 6a: the circular blank (Figure 6b) was previously painted in the peripheral region to reduce reflections and was clamped using a cylindrical cooler (to keep the temperature low in the central region) and a cylindrical holder.

The optimal values of FR and LP were validated on a 102 mm diameter blank. Temperature was monitored by means of two wire thermocouples (acquisition frequency set to 5 Hz) welded on the surface not irradiated by the beam and located at the centre of the specimen and at a radial position equal to 46 mm (labelled as TC1 and TC2 in Figure 6b, respectively). The laser spot centre was located at the radial position of 46 mm (in the case of the specimen in Figure 6a, only a portion of the spot irradiated the flange region); laser tests were monitored by the thermal image camera FLIR x6540sc (FLIR Systems, Wilsonville (OR), USA).

#### 2.4.2. Experimental Deep Drawing Tests

**Figure 7:**
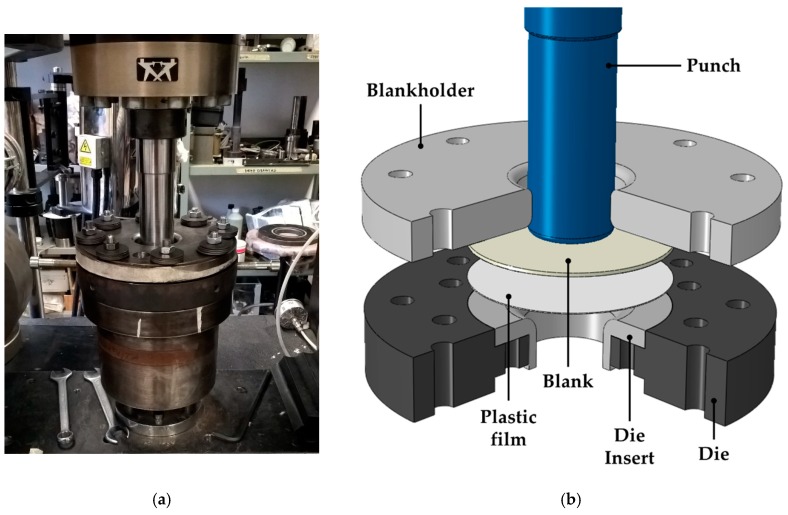
Deep drawing equipment: (**a**) experimental setup, (**b**) 3D representation of the components.

Experimental deep drawing tests were carried out adopting the laboratory scale equipment which was assembled on the same Instron machine used for the material characterization (Figure 7a).

The main components of the equipment are more clearly reported in the 3D representation of Figure 7b: during the deep drawing tests, the punch (diameter of 46 mm with a fillet radius of 3 mm) forced the blank to be deformed inside the cavity of the die, composed of a bigger disc and a modular insert (diameter equal to 50 mm, entry radius equal to 8 mm), while the blank holder was used to exert the pressure (Blank Holder pressure, BHp) for drawing in the material. All tests were carried out setting a punch speed equal to 6 mm/min and interposing a plastic film between the blank and the insert top surface to reduce friction; in addition, the blank portion in contact with the blank holder was sprayed with a Teflon-based lubricant.

## 3. Results and Discussion

### 3.1. Results from the Material Characterization

**Figure 8:**
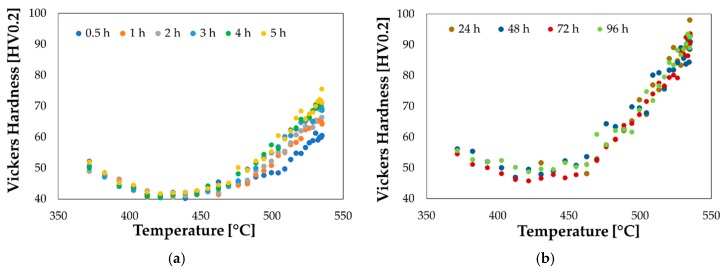
Evolution of the natural ageing phenomena: (**a**) from 0.5 h to 5 h; (**b**) from 1 to 4 days.

Gleeble experimental tests for investigating the achievement of the supersaturated condition were carried out setting the temperature at the TC2 location equal to 540 °C for a holding time equal to (0.6 h); soon after the heating, the specimen was cooled down by means of forced air and finally disassembled and maintained at room temperature. Evolution of the natural ageing was monitored by means of Vickers hardness measurements (Qness micro hardness tester Q10 A+, setting a load of 200 g and a dwell time of 15 s) along the longitudinal symmetry axis of the specimen at regular time intervals. Measured values have been plotted in Figure 8 as a function of the heating temperature, calculated in each point of the specimen longitudinal axis being known the parabolic distribution (Figure 1b) through the temperature acquisition by thermocouples.

As shown in Figure 8a, the measured hardness in the region close to the thermocouple TC2 (heating temperature of 540 °C) changed significantly during the first 5 h starting from the first recorded value equal to 55 HV, in accordance with data available in literature [43]. It is worthy of notice that, as it can be seen from the hardness distribution in Figure 8b, the natural ageing phenomena saturated almost completely after 24 h.

Such an investigation resulted to play a key role in the subsequent step aimed at designing the forming operations: circular blank had to be deep drawn soon after having been locally treated by laser heating, so that even a small evolution of the solutioned portion of the blank would not have compromised the outcome of the drawing step.

**Figure 9:**
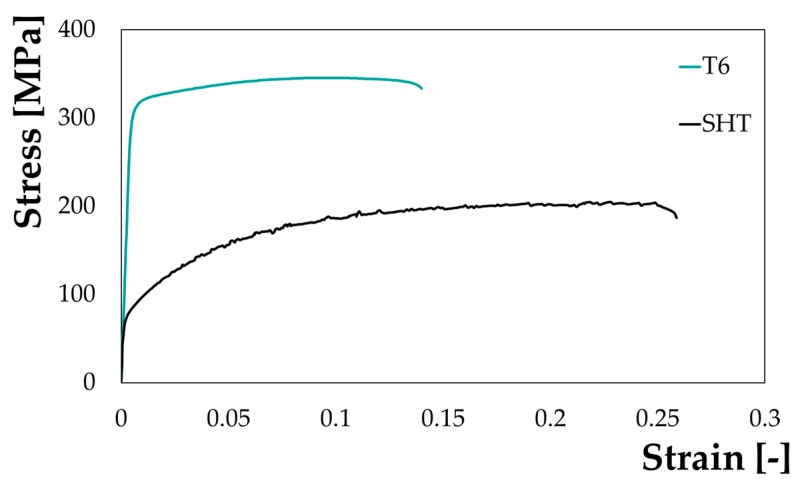
Stress strain curves at room temperature from the uniaxial tensile tests.

Material data from the tensile tests of the two investigated conditions are reported in terms of stress-strain curves in Figure 9: the specimen in the supersaturated condition (SHT, black bold curve) exhibited a sensibly higher strain at fracture and much lower strength (yielding stress about 70 MPa); the as received condition, on the other hand, showed a large mechanical strength (yielding stress higher than 300 MPa) and a limited fracture strain (less than 0.15). Stress-strain curves in Figure 9 resulted in good accordance with what reported by Cuniberti et al. [43], who registered a yield point of the same alloy in the fully solutioned conditions (1 h at 530 °C and then water quenched) around 75 MPa (fracture strain higher than 0.25) whereas the T6 condition (0.5 h at 180 °C) was characterized by a yielding point of slightly less than 300 MPa (fracture strain around 0.15).

Material behaviour in the SHT condition was characterized by a serrated flow: literature reports that, immediately after quenching, solute atoms are randomly distributed in solid solution in the Al matrix and interact with dislocations via a Cottrell cloud mechanism [44] assisted by a high vacancy concentration, leading to the observed serrated behaviour. With the proceeding of the natural aging, the solute atoms and vacancies form clusters, reducing the solute quantity in solid solution, thus explaining why the serrations disappeared in the T6 state [43].

The investigation of the material behaviour was completed by performing uniaxial tensile tests on the specimens previously subjected to LHT (Section 2.2.3). Due to the temperature decrease towards the specimen’s heads (the specimen was placed on a cooling ring), each point of the specimen experienced a different thermal history.

**Figure 10:**
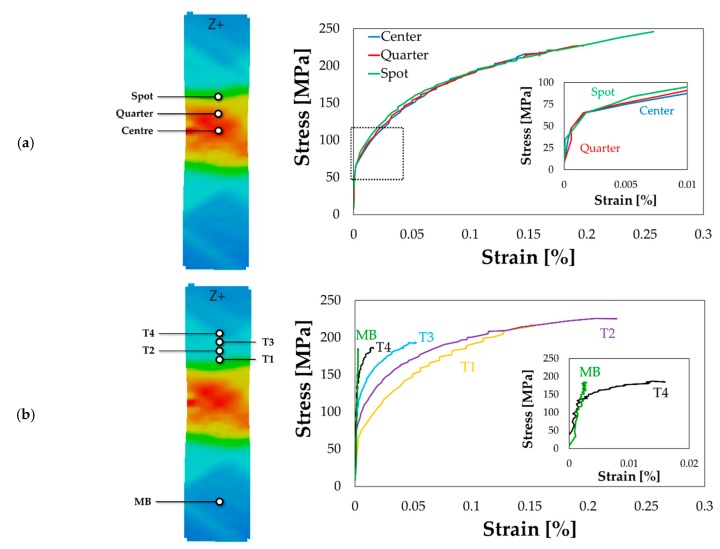
Uniaxial tensile test on the LHT specimen: flow curves from (**a**) the irradiated portion and (**b**) from the transition region and base material.

The uniaxial tensile test was carried out setting a crosshead speed of 6 mm/min and an acquisition frequency of the DIC cameras equal to 1 Hz. The adoption of the DIC system gave the big advantage of locally monitoring the strain evolution at any location of interest: in such a way, coupling the local strain information with the load data from the Instron machine, it became possible to extract several local flow curves from different locations within the same uniaxial tensile test. In the post-processing step, several points of interest were monitored, located: (i) in correspondence of the spot geometrical centre (labelled as “Centre” in Figure 10a), (ii) at one quarter of the spot dimension (labelled as “Quarter” in Figure 10a), (iii) at the end of the laser heated portion (labelled as “Spot” in Figure 10a), (iv) at four points located in the transition region immediately after the treated region with a step of 1.6 mm (labelled as “T1”, “T2”, “T3”, “T4” in Figure 10b) along the longitudinal axis of the specimen and (v) far from the treated region where the material was supposed not to be influenced by the laser heating (labelled as “MB” in Figure 10b).

For the sake of clarity, the calculated flow curves have been grouped in two different plots: Figure 10a shows the flow curves extracted by the irradiated portion of the specimen, while in Figure 10b flow curves from the transition region and base material have been plotted.

Flow curves in Figure 10a demonstrate that the whole portion irradiated by the laser beam was uniformly solutioned, being the curves perfectly overlapped (the detail in the plot further demonstrates that the points irradiated by the laser beam yielded at the same stress level). On the other hand, moving along the transition region from the treated portion to the base material, the laser heating was less effective and, as shown in Figure 10b, the material behaviour was characterized by progressively higher resistance. If the flow curve labelled as “MB” is considered (the detailed view compares more clearly the “MB” green curve with the “T4” black one), no yielding point could be distinctly identified suggesting that the material remained in the elastic region.

**Figure 11:**
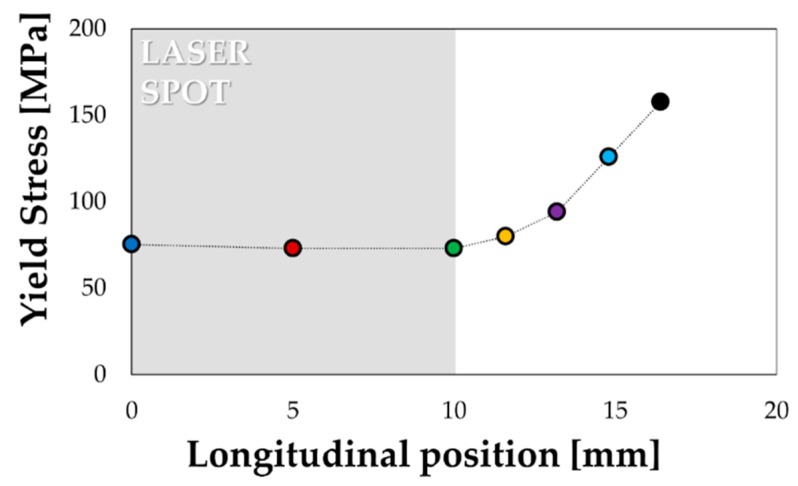
Distribution of the material yield stress along the specimen longitudinal axis.

A further confirmation came when plotting the yield stress of the monitored points as a function of their position along the specimen longitudinal axis (Figure 11). Yield stresses calculated within the treated region (grey box in Figure 11 refers to half spot, being the position 0 coincident with the geometrical centre of the spot) showed a good accordance (70 MPa) with the results of the uniaxial tensile test on the specimen in the SHT condition (Figure 9), while the transition region interested approximately 10 mm.

**Figure 12:**
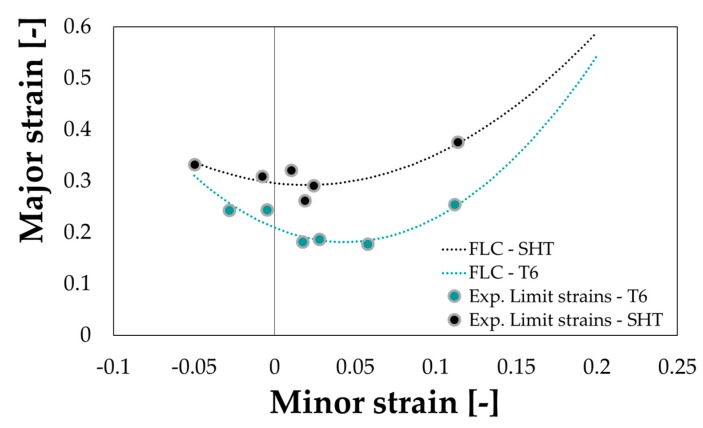
AA6082 Forming Limit Curves.

As regarding the strain behaviour from formability tests, Figure 12 clearly confirms how the peak hardening conditions remarkably limited the material formability if compared to the material behaviour in the SHT condition.

### 3.2. Solution Heat Treatment via Local Laser Heating

**Figure 13:**
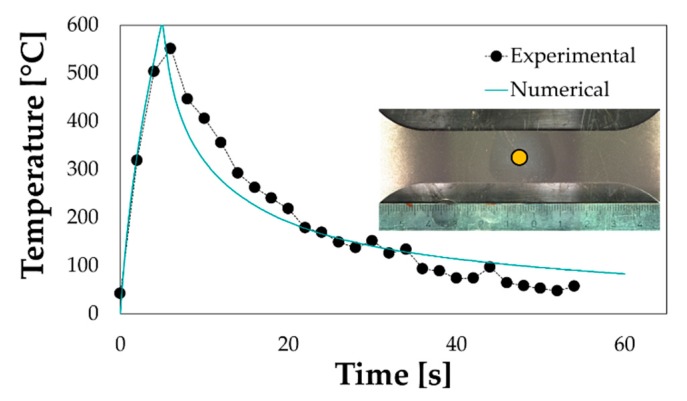
Tuning of the FE thermal model (the detailed view of the treated specimen is also reported).

The optimal values of the HTCs and of the absorption coefficient were chosen as the values able to minimize the discrepancy between numerical and experimental results (the monitored point is highlighted by a yellow circle). The fitting between numerical and experimental data using the thermal model after the tuning has been presented in Figure 13.

The optimized model parameters could be transferred to the FE model for designing the laser heating of the flange portion of the circular specimens to be subsequently drawn at room temperature. The movement of the laser spot along a circular track was implemented by means of the DFLUX user subroutine (Fortran).

**Figure 14:**
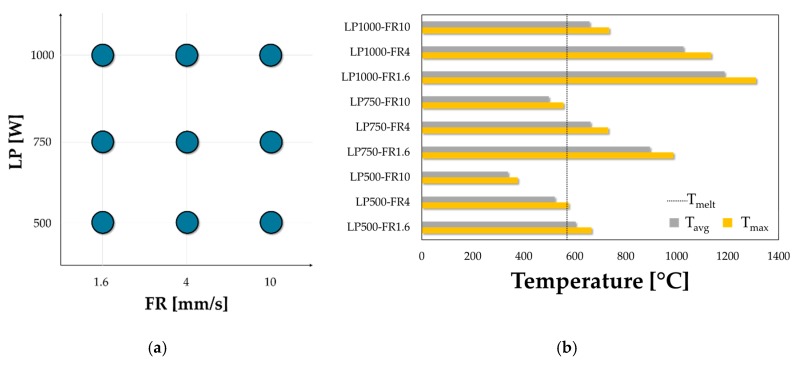
LHT numerical simulation (FR = 4 mm/s): temperature distribution after (**a**) half and (**b**) at the end of the heating.

As previously mentioned, the numerical simulations were arranged according to a Full Factorial plan investigating three levels of LP and FR, as shown in Figure 14a. Numerical results were analysed in terms of maximum temperature (T_max_) and average temperature (T_avg_) along the radial extent irradiated by the laser beam: the bar chart in Figure 14b suggests that the average temperature reached within the treated portion were comparable to those of Figure 13 only for two simulated conditions, namely LP750-FR10 and LP500-FR4.

Nevertheless, in the latter condition, the maximum temperature overcame the melting point of the alloy (indicated by a vertical black dotted line in Figure 14b); in addition, due to the increased heating time (lower FR value), the central portion of the blank could be affected by the treatment despite the presence of the cooler, thus obtaining a non-optimal distribution of properties. It was then concluded that the LP750-FR10 represented the best heating strategy to be experimentally applied.

**Figure 15:**
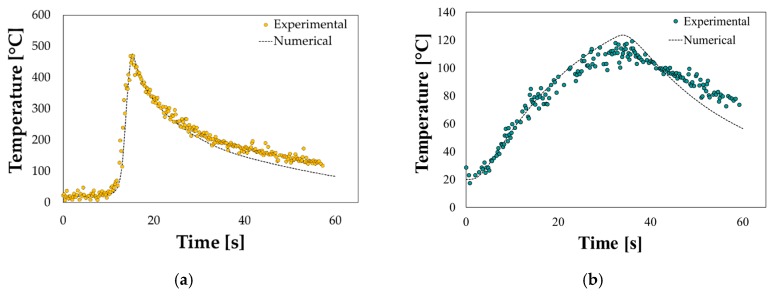
LHT of the circular blank: numerical vs. experimental comparison at the (**a**) thermocouple 1 (TC1) and (**b**) TC2 locations.

The numerically determined optimal combination of the LHT parameters was experimentally validated via experimental tests: as shown in Figure 15a,b, a good correspondence between the numerical temperature evolution and the data acquired from the thermocouples (TC1 and TC2) could be obtained.

**Figure 16:**
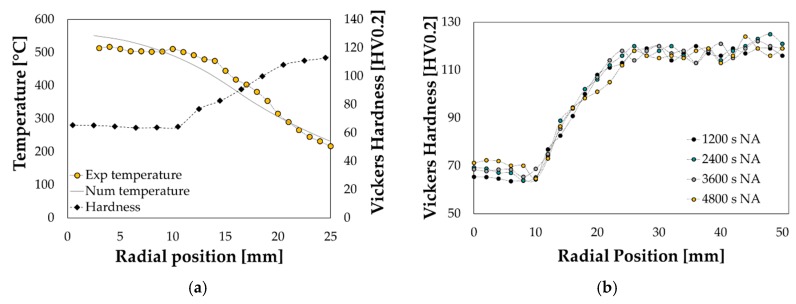
LHT on circular specimen: (**a**) hardness measurement and (**b**) progress of the natural ageing.

In addition, the temperature distribution over the blank was monitored by the thermal imaging camera FLIR x6540sc; after the cooling down to room temperature, the specimen was subjected to hardness measurements along the radial path to evaluate the effectiveness of the laser treatment. Temperature data plotted in Figure 16a suggest that:

(i) the temperature across the portion irradiated by the laser beam (the *0* radial position is located approximately at the blank outer diameter) was quite uniform and close to 500 °C; moreover, the numerical model (grey bold line) was accurate enough in reproducing the experimental temperature distribution (curve with yellow circle markers).
(ii) the laser heating was effective being the hardness values in the treated region similar to what measured during the tests on Gleeble (see Section 3.1) and sensibly lower than those measured close to the blank centre (kept in the T6 condition thanks to the continuous contact with the cooler).

Moreover, as shown in Figure 16b, the gradual increase of the hardness within the first 10 mm (up to more than 70 HV after an exposure of 4800 s at room temperature) was a clear evidence of the natural ageing (NA) occurrence. All the additional laser treatments for the subsequent experimental deep drawing trials at room temperature were thus carried out setting LP to 750 W and FR to 10 mm/s.

### 3.3. Preliminary Deep Drawing Tests at Room Temperature

**Figure 17:**
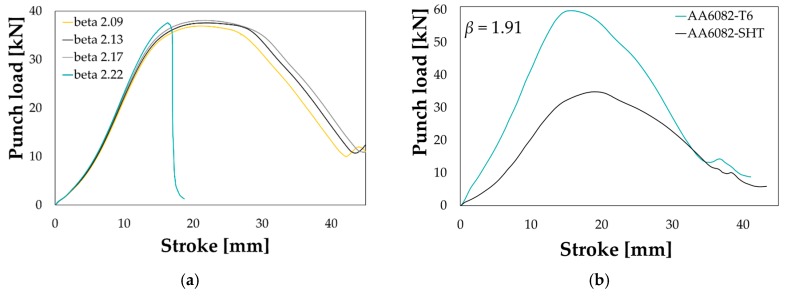
Punch load-stroke curve: effect of (**a**) the initial blank diameter (SHT condition, BHp = 1%) and (**b**) the initial material condition (*β* = 1.91, BHp = 1.5%).

Preliminary deep drawing trials were carried out to evaluate the limits in terms of formability of the two investigated material conditions, i.e., the T6 and the SHT. All experimental tests were monitored and reported in Figure 17 in terms of punch load and punch stroke, effective in quantifying the influence of the main process parameters.

As reported in Figure 17a, the effect of the initial blank diameter is in accordance with the theory: when setting the same friction condition (interposition of the plastic film) and the same BHp level (1% of the material yield stress), the peak punch load increased according to the drawing ratio β (it is the ratio between the initial blank diameter and the punch diameter); when β increased too much, the strain condition became too critical and the rupture occurred (green curve *“beta 2.22”*). As expected, the initial material conditions had a big influence on the drawing operation (see comparison in Figure 17b for a *β* value equal to 1.91). If setting the same friction conditions (plastic film in both tests) and applying the same BHp (1.5% of the yield stress), the peak load needed to successfully draw the material in the T6 conditions was almost twofold higher than the one needed to draw the material in the SHT conditions. Experimental deep drawing tests were also carried out, in both the investigated conditions, varying the BHp level for the same initial blank diameter, being the load exerted by the blank holder one of the most influencing parameter in the evaluation of the material drawability.

**Figure 18:**
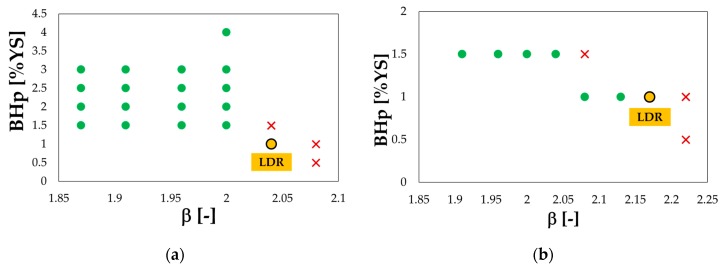
AA6082 experimental deep drawing tests: (**a**) T6 and (**b**) SHT condition.

Results of the preliminary experimental deep drawing tests were arranged in β—BHp plots and are reported in Figure 18a,b: green dots refer to a sound drawn component, while red crosses to occurred ruptures. 

In the case of the T6 condition, it can be noted that, for β values lower than 2, the applied BHp had a negligible effect on the process outcome: the achieved LDR was slightly higher than 2, in accordance with data reported in literature [45]. On the other hand, the achieved LDR slightly increased in the SHT condition (2.17) thanks to the improved material formability.

### 3.4. Results from the Process Optimization

**Figure 19:**
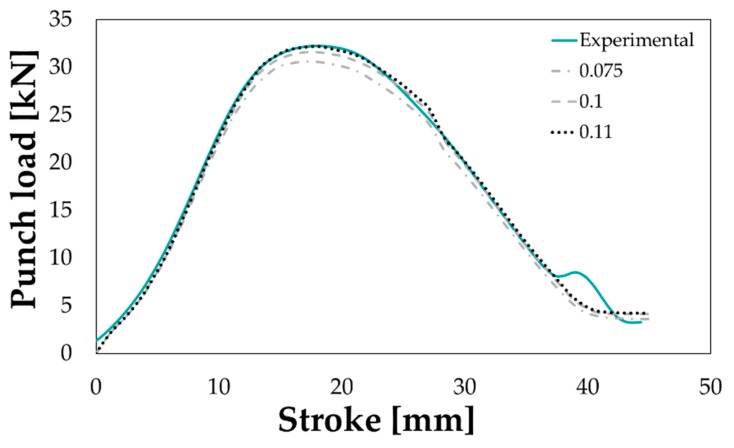
Comparison between the experimental punch load-punch stroke curve and the correspondent numerical ones obtained changing the friction coefficient in the range 0.075–0.11.

The tuning of the FE model adopted for the simulation of the Deep Drawing test was performed using again the inverse analysis approach. In particular, experimental data in terms of punch load-punch stroke curve were used as target data in order to determine the most effective value of the friction coefficient between the blank and the tools. The friction coefficient was iteratively changed and the discrepancy between the experimental curve and the one calculated by the numerical model was evaluated for each iteration. As reported in Figure 19, the best fitting between the experimental and the numerical data could be obtained setting the friction coefficient to 0.11.

**Figure 20:**
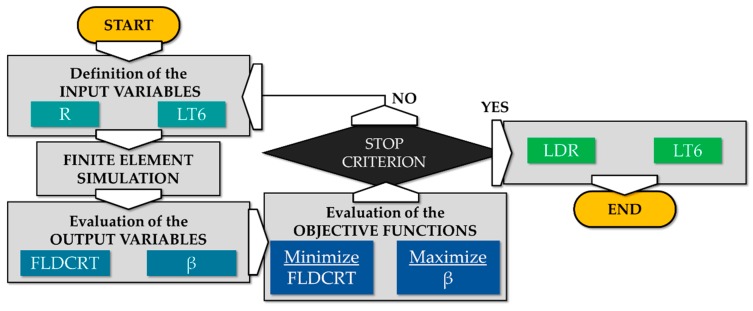
Basic principle of the optimization procedure.

The aim of the process optimization was to determine the optimal extent of the blank portion to be kept in the purchasing conditions (L_T6_) to increase as possible the drawable blank radius (R). The basic principle of the optimization procedure is schematically depicted in Figure 20.

Results from the FE-based process optimization have been reported in terms of history charts, useful to monitor the evolution of the defined input variables as the optimization proceeded: feasible designs are indicated by a green circle while the unfeasible ones—i.e., those characterized by a maximum value of the FLDCRT variable higher than 1—by red triangles.

**Figure 21:**
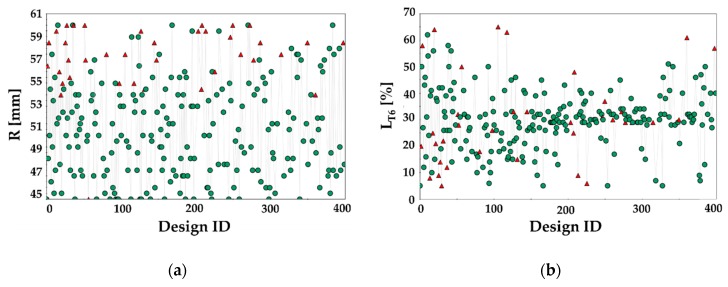
Results of the optimization procedure. History chart of the input parameter (**a**) R and (**b**) L_T6_.

According to the chart in Figure 21a, the optimization predicted a maximum sound diameter equal to the upper bound of the R variable (60 mm): an LDR value of 2.61 resulted then remarkably higher (more than 20%) than the limits experimentally achieved in both the material conditions. It is worthy of notice that, as shown by the history chart in Figure 21b, most of the feasible designs were characterized by a value of the L_T6_ parameter ranging from 30% to 40%. 

**Figure 22:**
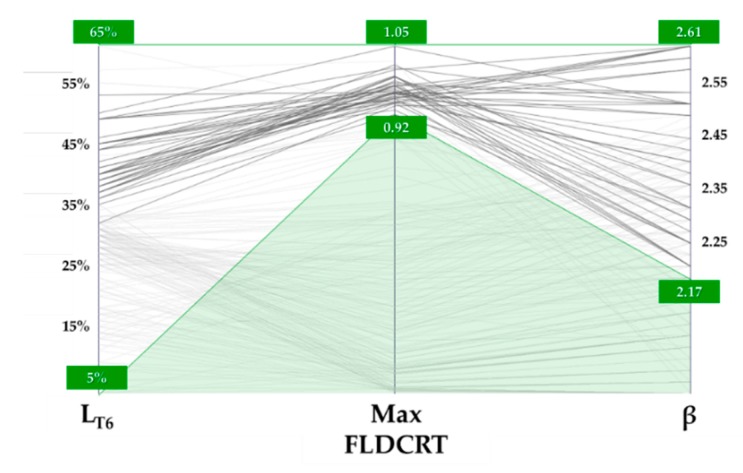
Optimization results: parallel coordinate chart.

That is even more evident if filtering the optimization results arranged in the parallel coordinate chart, where each design is represented by a polyline connecting the input variables to the defined output, as shown in Figure 22. If an LDR value higher than 2.17 (the limit reached in the SHT conditions) had to be achieved and the strain severity had to be kept below the tearing condition (FLDCRT < 1), the portion to be preserved from the heat treatment had to cover at least 35% of the whole blank.

### 3.5. Experimental Validation of the Numerical Optimization

**Figure 23:**
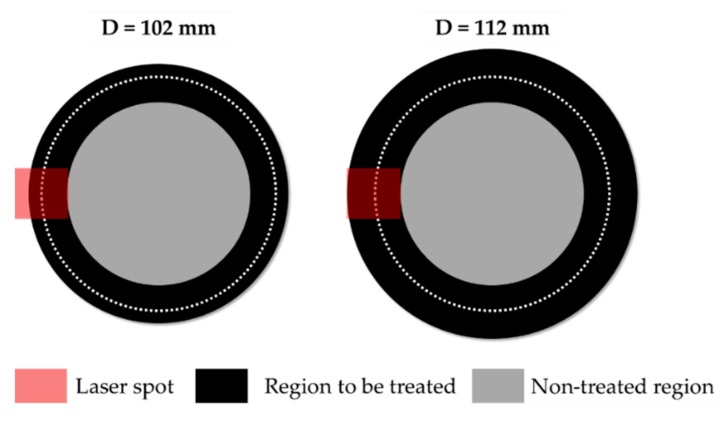
LHT tests. A portion of the spot irradiating the surface with the increasing blank diameter.

The deep drawing experimental tests for validation purposes were carried out immediately after having performed the LHT (to minimize the natural ageing phenomena). Laser treatments on all the investigated blanks were carried out locating the spot centre at an initial radial position equal to 46 mm and setting the FR to 10 mm/s while keeping LP constant (750 W). Each LHT test was replicated twice, so that the first sample could be used to evaluate the effectiveness of the treatment in terms of hardness distribution whereas the second one could be subsequently deep drawn. The local treatment operations started from an initial blank diameter equal to 102 mm (the maximum drawn dimension in the SHT condition) and, according to the scheme in Figure 23 (left image), only a portion of the spot irradiated the blank surface: in that condition, assuming that the whole power was absorbed by the material, the blank was irradiated by a specific power approximately equal to 2.4 W/mm^2^.

**Figure 24:**
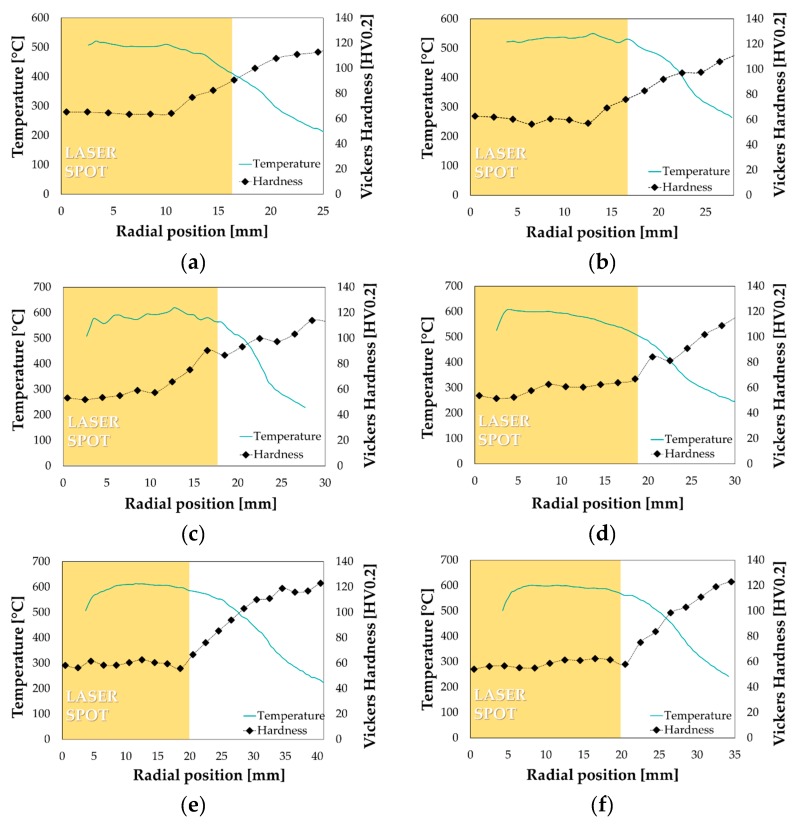
Temperature distribution and hardness measurement after LHT: (**a**) D = 102 mm, (**b**) D = 104 mm, (**c**) D = 106 mm, (**d**) D = 108 mm, (**e**) D = 112 mm, (**f**) D = 114 mm.

When increasing the initial diameter, since a progressively greater portion of the spot irradiated the blank, the laser power was accordingly increased to keep the irradiance constant. Experimental temperature distributions from the thermal camera combined with the subsequent hardness measurement along the same radial investigation path have been shown in Figure 24 (from a to f).

It can be noted that the increase of the laser power according to the blank diameter led to an almost uniform temperature distribution (higher than 500 °C) in the blank region irradiated by the spot (highlighted by the yellow box); in addition, the treated portion was effectively solutioned as proved by the hardness evolution along the radial direction.

The subsequent experimental deep drawing tests at room temperature were carried out setting the punch speed equal to 6 mm/min and adopting a value of the BHp equal to 1.5%, as FE simulations suggested. 

**Figure 25:**
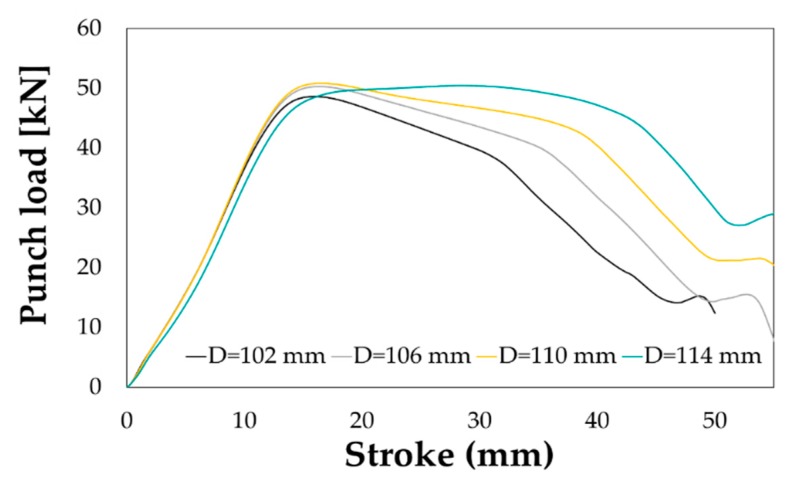
Deep drawing on LHT specimen. Effect of the blank diameter.

The increasing blank diameter, as already shown in Figure 17a, had an appreciable influence on the shape of the punch load curve as well as on its peak value (see Figure 25).

An LDR equal to 2.48 (blank maximum diameter equal to 114 mm) was achieved, confirming the advantages of the local heat treatment in enhancing the formability of the alloy at room temperature. 

**Figure 26:**
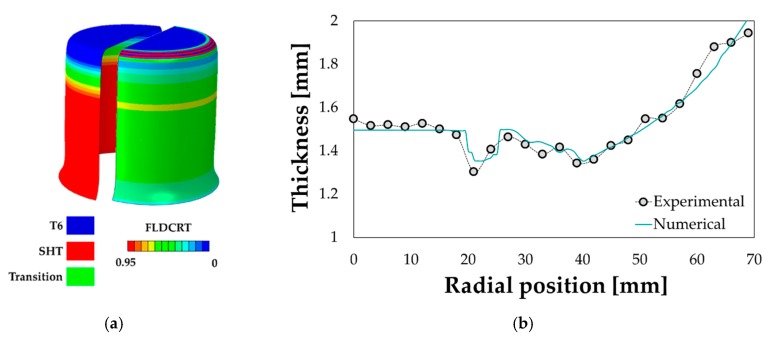
Validation of the optimization prediction: (**a**) numerical distribution of material properties and strain severity (FLDCRT), (**b**) numerical vs. experimental thickness distribution.

The numerical best design from the optimization round slightly overestimated the final LDR (2.61 vs. 2.48) of about 5% that was considered acceptable. As a further confirmation, if comparing the experimental results from the maximum drawn diameter with the correspondent numerical simulation from the optimization round, the successful drawing was numerically predicted if the blank portion kept in the T6 condition covered 46% of the whole blank, which means a radial extent of the fully solutioned portion slightly higher than 20 mm exactly equal to what experimentally measured (see the hardness profile in Figure 24f). It is worthy of notice that, as visible from the contour maps in Figure 26a, the most severe strain condition (FLDCRT) occurred close to the punch radius but, thanks to softening of the flange region and an improved material drawing, rupture could be avoided.

Despite the small discrepancy in terms of LDR, the properly tuned FE model (especially in terms of friction coefficient) resulted to be accurate even in predicting the final thickness distribution along the drawn cup (see Figure 26b).

## 4. Conclusions

In this work the effectiveness of tailoring the material properties by a short-term local treatment to improve the AA6082 formability at room temperature has been investigated by means of a numerical/experimental approach. 

The preliminary local laser heating of the dog bone specimen, carried out for characterization purposes, resulted to be effective in defining the thermal boundary conditions of the FE model and properly designing the local treatment of the circular blanks to be subsequently drawn at room temperature. In addition, the subsequent uniaxial tensile tests on the LHT specimens, thanks to the DIC assistance, provided several local flow curves to be implemented within the 2D axisymmetric deep drawing FE model. 

Even if the deep drawing model was simple and low time consuming, being coupled with the optimization platform modeFRONTIER, it allowed to determine the best LHT conditions in terms of extension of the treated region: the results of the optimization round, based on the creation of 20 generations starting from an initial population of 20 individuals, suggested that at least 35% of the blank had to be kept in the T6 condition to improve the LDR up to 2.61, slightly higher (less than 5%) than the limit experimentally achieved. 

Despite the simple formulation in the material constitutive equation (the isotropic Von Mises model was used for the material yield condition), the registered discrepancy was considered acceptable and a further confirmation of the accurateness of the optimization results came from the prediction of the final thickness distribution of the drawn cup in the experimental LDR condition. 

The presented results can be considered remarkable since the optimized distribution of material properties led to an evident improvement of the material drawability not only with respect to the T6 condition (+22%)—which was quite reasonable because of the limited formability due to the peak hardening condition—but also to the more formable SHT condition (+14%); the obtained gain in the material formability at room temperature resulted in line with data in literature, according to which an optimized treatment strategy is able to increase the LDR of a 6000 Al alloys from 2 up to 2.5 [46,47].

The achieved results confirm the effectiveness of the proposed approach in the case of a relatively simple manufacturing process, but, at the same time, open the way to its adoption for the manufacturing of a broad range of components, even characterized by high complexity (in terms of shape and/or mechanical characteristics). In fact, the laser heating allows to selectively and very flexibly change the material properties, which is a big advantage in the early stages of the process design, when the heating strategy has to be properly defined and optimized. Next steps of the research will be aimed at evaluating effective strategies able to combine the maximization of both the formability and the shape accuracy of complex shaped real parts.

## Figures and Tables

**Table 1:** Chemical composition of the AA6082 under investigation.

Al%	Si%	Fe%	Cu%	Mn%	Mg%	Cr%	Zn%	Ti%
Bal	0.7–1.3	≤0.5	≤0.1	0.4–1	0.6–1.2	≤0.25	≤0.2	≤0.1

**Table 2:** Definition of the input variables and their variation ranges.

Input Variable	Lower Bound (LB)	Upper Bound (UB)
L_T6_ [%]	5	65
R [mm]	45	60
BHp	Constant and equal to 1.5% of the material yield stress	

## List of Symbols and Acronyms

***LDR***	Limit Drawing Ratio
***FLC***	Forming Limit Curve
***FLDCRT***	output variable from the FLC-based damaged criterion (Forming Limit Diagrams CRiTerion)
***SHT***	Solution Heat Treatment
***BHp***	BlankHolder pressure (action exerted by the blankholder, in MPa, during the deep drawing experimental trials)
***HTC***	Heat Transfer Coefficient
